# Screening and Identification of a Chicken Dendritic Cell Binding Peptide by Using a Phage Display Library

**DOI:** 10.3389/fimmu.2019.01853

**Published:** 2019-08-06

**Authors:** Sunting Ma, Xinyuan Qiao, Yigang Xu, Li Wang, Han Zhou, Yanping Jiang, Wen Cui, Xuewei Huang, Xiaona Wang, Lijie Tang, Yijing Li

**Affiliations:** ^1^College of Veterinary Medicine, Northeast Agricultural University, Harbin, China; ^2^Heilongjiang Key Laboratory for Animal Disease Control and Pharmaceutical Development, Harbin, China; ^3^Key Laboratory of Veterinary Biological Engineering and Technology, Ministry of Agriculture, Institute of Veterinary Medicine, Jiangsu Academy of Agricultural Sciences, Nanjing, China

**Keywords:** chicken dendritic cells-binding peptides, probiotic bacteria, oral immunization, vaccine delivery, infectious bursal disease virus

## Abstract

Dendritic cells (DCs), as antigen-presenting cells, can initiate adaptive immune responses efficiently. Although the DC-targeting strategy has attracted more attention, relevant studies on chicken are rare. Here, specific chicken bone marrow DC-binding peptides were selected using a phage display peptide library and confirmed through ELISA, flow cytometry, fluorescence microscopy, and laser confocal microscopy. The peptide candidate SPHLHTSSPWER, named SP, was fused to the infectious bursal disease virus (IBDV) structural protein and protective antigen VP2. *In vitro*, the expression of DC markers (CD80, CD83, CD86, DEC205, and MHCII) and some cytokines (IFN-γ, IL-12, TNF-α, IL-1β, IL-6, and CXCLi1) by VP2-SP-stimulated DCs was significantly higher than that by DCs treated with the VP2-control peptide at 4 h (*p* < 0.001). In addition, an oral vaccine targeting DCs was generated using chicken-borne *Lactobacillus saerimneri* M11 (*L. sae* M11) to deliver VP2 fused with SP. Anti-IBDV mucosal and humoral immune responses were induced efficiently via oral administration, resulting in higher protective efficacy in the VP2-SP group than the VP2 group. Therefore, chicken DC targeting of IBDV protective antigen VP2 delivered by *L. sae* provides effective immune protection in chicken. Our study may promote research on the DC-targeting strategy to enhance the effectiveness of chicken vaccines.

## Introduction

With the increase in poultry production in recent years, preventing avian disease has become a concern worldwide. New and improved vaccines for avian diseases are urgently needed. Infectious bursal disease (IBD), an immunosuppressive disease, caused by infectious bursal disease virus (IBDV) has always been a major concern for poultry farmers. IBDV can infect immature B-lymphocytes in the bursa of Fabricius (1) and result in vaccination failure and susceptibility of chickens to pathogens (2). Both maternal immunity and active immunity can protect chicks against IBDV infection. Currently, live-attenuated and genetically engineered viral vectors that express the IBDV surface protein VP2 can be used to produce active immunity in chicks (3). VP2 protein is a structural component of the capsid and protective antigen containing neutralizing epitopes. In addition, subunit vaccines, DNA vaccines, virus-like particle vaccines, and lactic acid bacteria vaccines are constantly evolving as vaccine candidates (4, 5).

In the search for more effective candidates for treatment and prevention of diseases, dendritic cell (DC) targeting strategies have been proposed. DCs are specialized antigen-presenting cells that can capture, process, and present antigens to native T cells to initiate primary immune responses (6). Because of the unique capacity mentioned above, DCs are usually utilized as the target of the antigen for generating efficient and strong immune responses, especially in the mammalian antitumor field (7). One approach involves autologous DCs loaded with antigens *ex vivo* being re-injected into patients, and the other one targets DCs *in situ* by conjugating the vaccine with DC receptor-specific monoclonal antibodies (8, 9). There is also an *in situ* approach of targeting antigens to DCs using nanoparticles to encapsulate antigens and adjuvants (10, 11). A decade ago, specific DC-binding peptides screened by a phage display peptide library were identified (12). Subsequently, oral mucosal vaccines targeting DCs were proposed, involving *Lactobacillus* delivering protective antigens fused with specific DC-targeting peptides to DCs (13). The serum anti-antigen IgG titers, neutralizing antibodies, and the levels of IgA were all comparable with the historical adjuvanted vaccine administered subcutaneously (s.c.) (14–16). Additionally, the *Lactobacillus* vaccine has tremendous advantages such as weak immunogenicity, similar to the immune-stimulating adjuvant, initiating both mucosal, and systemic immune responses, improving practicality for mass vaccination, and ease of production and administration (17–19).

Chicken DCs have similar function to those in mammals, although the most responsive avian antigen-presenting cells (APCs) are still unknown (20). However, previous studies have demonstrated that compared to mammals, the chicken has a different repertoire of immunity genes, molecules, cells, and tissues. Many immunity genes such as chemokines, chemokine receptors, and Toll-like receptors are different between the chicken and mammals. The chicken lacks lymph nodes, but has the bursa of Fabricius and cecum tonsil, which are absent in mammals (21, 22). The research on avian immunity is relatively lagging compared with the mouse, human, and swine, which limits the development of an avian DC targeting strategy.

As a result of the difference between mammals and chickens, the mammalian ligands to DCs have some limitations when applied in chickens. In order to select chicken DC-targeting ligands, biopanning was carried out in the phage display peptide library on chicken bone marrow-derived DCs (chBM-DCs) in this study. At the same time, we used *Lactobacillus saerimneri* (*L. sae*), which has the ability to colonize the chicken intestine, as the delivery vector to reduce the inflammatory reaction from heterologous *Lactobacillus*. IBDV VP2 protein was chosen as a protective antigen. VP2 with or without chBM-DC-binding peptide was constitutively expressed on the surface of *L. saerimneri*, and the immunogenicity and protective efficacy were evaluated.

## Materials and Methods

### Virus, Bacteria, Plasmids, and Cell Line

Highly virulent IBDV isolated from the sick chick embryo allantoic liquid, cell-adapted IBDV strain, and DF-1 cells (chicken embryo fibroblasts) was maintained in our laboratory. *L. saerimneri* M-11 was isolated from 20-day-old chicken cecum and cultured in de Man, Rogosa, and Sharpe medium (MRS; Hopebol, Qingdao, China) without shaking. The constitutive expression plasmid pPG-T7g10-PPT, previously constructed by our laboratory, contained the HCE strong constitutive promoter, T7g10 transcriptional enhancer, pgsA anchor from *Bacillus subtilis* for stabilizing the heterologous protein in the cell membrane (surface-displaying), enhanced green fluorescent protein (eGFP), and the rrnBT1T2 terminator (23).

### Isolation and Validation of chBM-DCs

Marrow obtained from femurs and tibias of 4–6-week-old broiler chicks was washed twice with sterile phosphate buffer saline (PBS), resuspended in PBS, loaded in an equal volume of Histopaque-1119 (Sigma-Aldrich, St. Louis, MO, USA), and centrifuged at 1,200 × *g* for 30 min. Cells at the interface (chBM-DCs) were then collected as previously described (24) and seeded at 10^6^ cells mL^−1^ in 6-well plates containing Roswell Park Memorial Institute-1,640 supplemented with 1 U mL^−1^ penicillin and streptomycin, 10% fetal calf serum (Gibco, Grand Island, NY, USA), 50 ng mL^−1^ recombinant chicken granulocyte macrophage colony stimulating factor (Abcam, Cambridge, UK), and 25 ng mL^−1^ interleukin-4 (Kingfisher, Saint Paul, MN, USA) at 37°C and 5% CO_2_ for 6 days. Three-quarters of the medium were replaced with complete medium every 2 days. CD11c and MHCII expressed on the surface of DCs on the 6th day and CD40 and CD86 expressed on the surface of LPS-stimulated DCs on the 7th day were analyzed using flow cytometry.

### Screening of chBM-DC-targeting Peptides by Phage Display

The Ph.D.-12 phage display library (NEB, Beijing, China) displaying linear 12-mer random peptide at the N-terminus of P III protein of bacteriophage M 13 was applied to screen the DC-targeting peptide. First round: phages (2 × 10^11^) were added into chBM-DCs for 30 min at 4°C. The cell suspensions centrifuged at 600 × *g* for 8 min were resuspended in PBS containing 1% bovine serum albumin (BSA) and 0.05% Tween-20. After repeating the washing step three times, the number of phages bound to DCs was evaluated by the phage-plaque assay. The phages were amplified in *Escherichia coli* ER 2,738 for the next round of biopanning. Second-four rounds: phages were incubated with marrow cells and unbound phages were added into chBM-DCs for 15 min at 4°C, and then the procedures described above were repeated. After the fourth round of biopanning, individual phage-plaques were randomly selected and amplified separately. The nucleotide sequence of each phage extracted with the Phage DNA Isolation Kit (EasyExtraction, Beijing, China) was determined with −96 g III primer, and the sequence was translated into a peptide sequence.

### Determination of Binding Ability of Selected Phages With ELISA, Flow Cytometry, Fluorescence Microscope, and Laser Confocal Microscopy

- ELISA
  Marrow cells and chBM-DCs were fixed on 0.03 mg/ml polylysine-coated 96-well plates for 30 min at 25°C. Cells were blocked with the PBS (2% w/v BSA) for 30 min at 4 °C. Phage clones (10^10^ pfu/ml) were added to wells and incubated at 4°C for 20 h. PBS and wild M13 phages were used as a negative control. Cells were washed three times with PBST and then incubated with the goat anti-M13 bacteriophage polyclonal antibody (diluted 1:1,500 in 2% BSA, Sino Biogical, Beijing, China) at 37°C for 40 min. Subsequently, the horseradish peroxidase (HRP)-labeled rabbit anti-goat IgG antibody (diluted 1:4,000, ZSGB Biotech, Beijing, China) was incubated with the cells. Finally, the cells were washed with PBST and color was developed using a 3, 3′, 5, 5′-tetramethylbenzidine enzyme substrate substrate, and the absorbance at OD_450_ was measured.
- Flow cytometry
  The candidate peptides were synthesized using Fmoc protocols and purified with high-performance liquid chromatography (purity was >95%) by Xinghao Biotech (Wuhan, Hubei). The C-terminal was labeled with fluorescein isothiocyanate (FITC). FITC-labeled peptide (25 μg) was incubated with 10^6^ chBM-DCs at 4°C for 10 min. After being washed three times with PBS, cells were subjected to flow cytometry (BD Biosciences, San Jose, CA, USA). Experiments were repeated three times.
- Fluorescence microscopy and laser confocal microscopy
  To further evaluate the localization of peptides binding to chBM-DCs and monocytes, cells were all plated on coverslips overnight. Cells were fixed with polylysine on coverslips as described previously, washed, and incubated with 25 μg FITC-conjugated peptides for 20 min at 37°C. After being washed three times with PBS, slides were stained with 1,1'-dioctadecyl-3,3,3′,3′-tetramethylindocarbocyanine perchlorate (DIL) membrane probe (Beyotime, Haimen, China) for 10 min at 37°C and observed using a ZOE fluorescence microscope (Bio-Rad, CA, USA) and LSCM (model LSM510 META; Zeiss, Germany).

### Fusion of DC-Peptide to VP2

**Figure 1:**
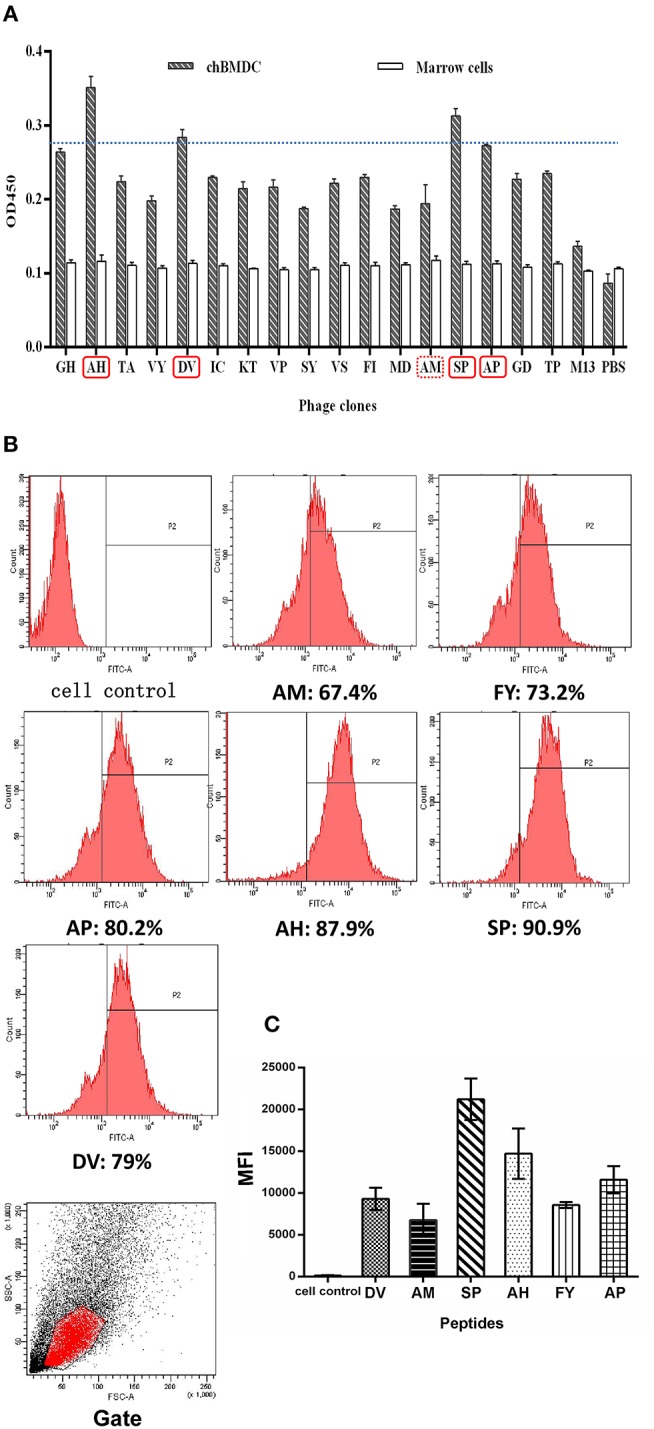
Ability of peptides to bind to chBM-DCs was analyzed by phage ELISA and flow cytometry. Phage ELISA: peptides with better binding ability to chBM-DCs are labeled with a solid line **(A)**. Flow cytometry analysis: the counts and gate of FITC-labeled peptides binding to DCs **(B)**. The mean fluorescence intensities of FITC-labeled peptides binding to DCs **(C)**.

- *Escherichia coli* expression system
  The VP2 coding sequence (sequence ID:AF240686.1) was amplified from an IBDV cDNA using PCR. SP (SPHLHTSSPWER) and control peptide (PPWTHSESRLSH, rearranging the sequence of SP) were fused separately to the C terminus of VP2 using primers listed in Table 1. After restriction digestion, the PCR product was inserted into multiple cloning sites in plasmid pCold^TM^ TF. Recombinant VP2 fusion proteins were expressed in *E. coli* BL21 (DE3) after 24 h of induction using 1 mM isopropyl-D- thiogalactopyranoside at 15°C. Cell-free extracts were generated by sonication, and the recombinant fusion proteins were purified using an Ni-NTA Superflow Column (TransGen Biotech, Beijing, China).
- Construction of recombinant targeted *Lactobacillus* strains
  A recombinant expression plasmid was constructed as shown in Figure 1. The primers used in this study are listed in Table S1. Targeting peptide was fused to the C terminus of *vp2*. Thus, the vp2-SP fusion genes were cloned into the expression plasmid pPG-T7g10-PPT-eGFP to generate pPG-eGFP-vp2-SP. To construct the recombinant *Lactobacillus* strain, the recombinant plasmids were electrotransferred into *L. saerimneri* M-11 as described previously (21), giving rise to the recombinant strain pPG-eGFP-vp2-SP/M-11. In addition, pPG-eGFP-vp2-SP/M-11 was also generated. Then, the expressed protein was analyzed by western blotting as described previously (25). Mouse anti-eGFP (1:4,000), mouse anti-pgsA anchor polyclonal antibody (1:200), and IBDV VP2 monoclonal antibody (1:40), all kept in our lab, were used as the primary antibody. Furthermore, fluorescence of eGFP expressed by *Lactobacillus* was evaluated by flow cytometry. Briefly, 2 ml of recombinant *L*. *sae* M-11 was grown in MRS broth containing chloramphenicol (10 μg/mL) at 42°C until OD_600_ ≈ 0.3, harvested by centrifugation, washed twice with sterile PBS, and analyzed by flow cytometry.

**Table 1:** Sequence of primers.

**Primers**	**Sequence (5^**′**^−3^**′**^)**
F-vp2	***GGTACC***ATGACGAACCTGCAAGAT
R-v-sp	***GGATCC***TTA**ACGTTCCCATGGACTACTAGTATGAAGATGTGGTGA**^a^ TGAGCCACCGCCACC^b^*CACCTCCATGAAGTACTCGCG*
R-v-ctrl	***GGATCC***TTA**ATGTGAAAGACGTGATTCACTATGAGTCCATGGTGG**^a^ TGAGCCACCGCCACCC^b^ACCTCCATGAAGTACTCGCG

Restriction enzyme recognition sites used for cloning are shown in bold and italics;

a *Dendritic cell-targeting peptide (DCpep) and control peptide (in bold)*.

b *Flexible amino acids are shown with underline*.

### Analysis of Marker Genes and Cytokine Expression by chBM-DCs Assessed by Relative qRT-PCR

chBM-DCs (10^6^/mL) were incubated with 2 μg/mL purified protein expressed in the *E. coli* system (VP2-ctrl, VP2-SP, Tag) at 37°C for 4, 6, and 8 h. Then, total RNA from non-stimulated chBM-DCs and chBM-DCs incubated with purified protein was isolated using a commercial kit according to the manufacturer's instructions (Omega Bio-Tek, Norcross, GA, USA). Quantitative real-time PCR was performed using the SYBR Green PCR Master Mix (Roche, Shanghai, China). The primers used for qRT-PCR were designed based on target sequences reported previously and are shown in Table S1. The qRT-PCR was performed using a 7,500 Real-Time PCR system (Applied Biosystems) with the following cycle profile: 95°C for 10 min, followed by 40 cycles of 95°C for 15 s, and 60°C for 1 min. β-actin was amplified as an internal control. The threshold cycle value (Ct) was obtained for each sample. The relative expression of each target gene was measured relative to that of β-actin using the formula ΔΔCt = (Ct_(stimulated−DC, targetgene)_- Ct_(stimulated−DC, β−*actin*)_)- (Ct_(non−stimulated−DC, targetgene)_- Ct_(non−stimulated−DC, β−*actin*)_), to calculate the fold change (2^−ΔΔCt^).

### Immunization and Specimen Collection

One-week-old specific pathogen-free (SPF) chicks were purchased from Harbin Veterinary Research Institute in China and kept under SPF conditions with free access to standard water and diet. All animal procedures were approved by the Ethical Committee for Animal Experiments (Northeast Agricultural University, Harbin, China). Prior to oral administration, the recombinant *Lactobacillus* strains were cultured overnight in MRS medium, washed with sterile PBS, and resuspended at 5 × 10^9^ CFU in 200 mL in PBS. Chicks were randomly divided into four groups (30 chicks per group): PBS (40 chicks), pPG/M-11, pPG-VP2/M-11, and pPG-VP2-SP/M-11 groups. The immunization dosage was 5 × 10^9^ CFU and was administered on two consecutive days (days 1, 2). Booster immunizations were administered on days 11, 12, 22, and 23.

Immunized chickens (3 chickens in each group) were sacrificed to collect intestinal fluid and tracheal fluid on days 0, 7, 15, 21, 26, and 35 after immunization. Serum was collected on days 0, 7, 15, 21, 26, 32, 35, and 38. Samples of the intestinal tract were stored at −40°C until analysis.

One-week-old broiler chicks without maternal immunity were purchased from the Harbin Sheng Li chicken farm. Chicks were randomly divided into four groups (24 chicks per group): PBS (32 chicks), pPG/M-11, pPG-VP2/M-11, and pPG-VP2-SP/M-11 groups. The immunization dosage was 5 × 10^9^ CFU and it was administered on two consecutive days (days 1 and 2). Booster immunization was administered on days 11 and 12.

### ELISA Analysis of Antibody Levels and Cytokine Analysis

The levels of IgY in the sera and IgA in intestinal fluid and tracheal fluid were measured by ELISA. Polystyrene microtiter plates were coated overnight at 4°C with IBDV propagated on DF-1 cells, and the culture of DF-1 cells was used as a negative control for the antigen. After blocking with 5% skimmed milk, the collected samples were serially diluted in PBS, added in triplicate, and incubated at 37°C for 1 h. Then, goat anti-chicken IgY (KPL, MD, USA) or mouse anti-chicken IgA (Southern Biotech, Birmingham, AL), both diluted 1:3,000, were then added as secondary antibodies and incubated at 37°C for 1 h. Color was then developed using 3,3′,5,5′-Tetramethylbenzidine (Beyotime, Shanghai, China) as a substrate, and the absorbance at OD_450_ was measured.

Cytokines released into the blood before and after IBDV challenge were analyzed by using chicken IFN-γ, IL-2, IL-4, and IL-6 commercial kits (AndyGene, Beijing, China).

### Neutralization Assay

Briefly, sera from chickens on day 26 post-immunization were filtered and inactivated at 65°C for 30 min. The sera were serially diluted 2-fold (1:2, 1:4, 1:8, 1:16, 1:32, 1:64……), mixed with an equal volume of virus suspension (200 TCID50), and incubated at 37°C for 1 h. The intermixture was inoculated onto a DF-1 cell monolayer at 37°C in a 5% CO_2_ incubator. The positive serum control, negative serum control, virus control, and blank control were prearranged. Cytopathic effect was observed daily. The Reed-Muench statistical method was used to calculate the results (26).

### Challenge Experiment and Histopathological Examination

Virulent IBDV was used to evaluate the protective efficacy of oral immunization with the recombinant strains. Four groups of SPF chickens (15 per group) were orally challenged with very virulent IBDV at 35 days post-immunization, and the dose was 400 LD_100_ (absolute lethal dose) of chicken embryo. Chicken health was monitored daily for a 14-days observation period, and chickens that developed severe clinical symptoms were euthanized. The cumulative mortality of chickens in each group was recorded. At 14 days post-challenge, surviving animals were sacrificed and necropsy was performed. Bursa Fabricius to body weight (BBWR) was calculated by: (bursal weight/body weight) × 1,000 (27).

Four groups of broiler chickens (24 per group) were orally challenged with very virulent IBDV at 22 days post-immunization, and the dose was 350 LD_100_ of chicken embryo. Chicken health was monitored daily for a 14-days observation period, and chickens that developed severe clinical symptoms were euthanized. The cumulative mortality of chickens in each group was recorded. At 14 days post-challenge, surviving animals were sacrificed and necropsy was performed. Bursa Fabricius to body weight (BBWR) was calculated by (bursal weight/body weight) × 1,000.

### Statistical Analysis

Experiments were repeated three times, and the results are shown as the mean ± SE of three replicates per condition. Non-parametric (Mann-Whitney test) was used to analyze differences between groups in animal experiment and two-way analysis of variance (ANOVA) was used in other experiments. *p* < 0.05 was considered statistically significant, *p* < 0.01 was considered highly significant, and *p* < 0.001 was considered extremely significant.

## Results

### Generation and Characterization of chBM-DC-targeting Peptide

To screen and select peptide ligands that specifically target DCs, four rounds of phage display biopanning were performed, and phages unbound to marrow cells were added to DCs to reduce the possibility of non-specific binding. Table 2 shows phage recovery in each round tended toward stability, which was thought as evidence of our effective screening. In the fourth round, 168 phages were randomly selected for sequencing (Supplementary Figure 1). Of these, 54 phages displayed no peptides, as wild phages had stronger infectivity than phages displaying the peptide. Four phage isolates in Table S2 frequently appeared. This suggests that these four peptides may be more capable of binding to chBM-DCs.

**Table 2:** Determination of phage recovery rate.

**Round**	**Input phages (PFU)**	**Marrow cells-binding phages (PFU)**	**Output phages (PFU)**	**Recovery rate (output/input)**
1	2 × 10^11^		1.31 × 10^6^	6.55 × 10^−6^
2	1.86 × 10^11^	2.3 × 10^9^	1.7 × 10^6^	9.14 × 10^−6^
3	2.1 × 10^11^	3.4 × 10^9^	4.83 × 10^5^	2.3 × 10^−6^
4	1.45 × 10^11^	3.8 × 10^9^	2.15 × 10^5^	1.48 × 10^−6^

For further confirmation, we chose another 13 phages in the fourth round to evaluate binding ability together with phages mentioned above by using ELISA. Figure 1A shows that selected phages hardly bound to the marrow cells, as expected. According to the result of ELISA, besides SP, another three peptides (AH, DV, AP) with better binding potentiality were synthetized for further experiments. The result illustrated peptides that occurred once may also have binding ability that should not be ignored. Meanwhile, AM was chosen as a control and mammalian peptide (FY) was synthetized for reference.

**Figure 2:**
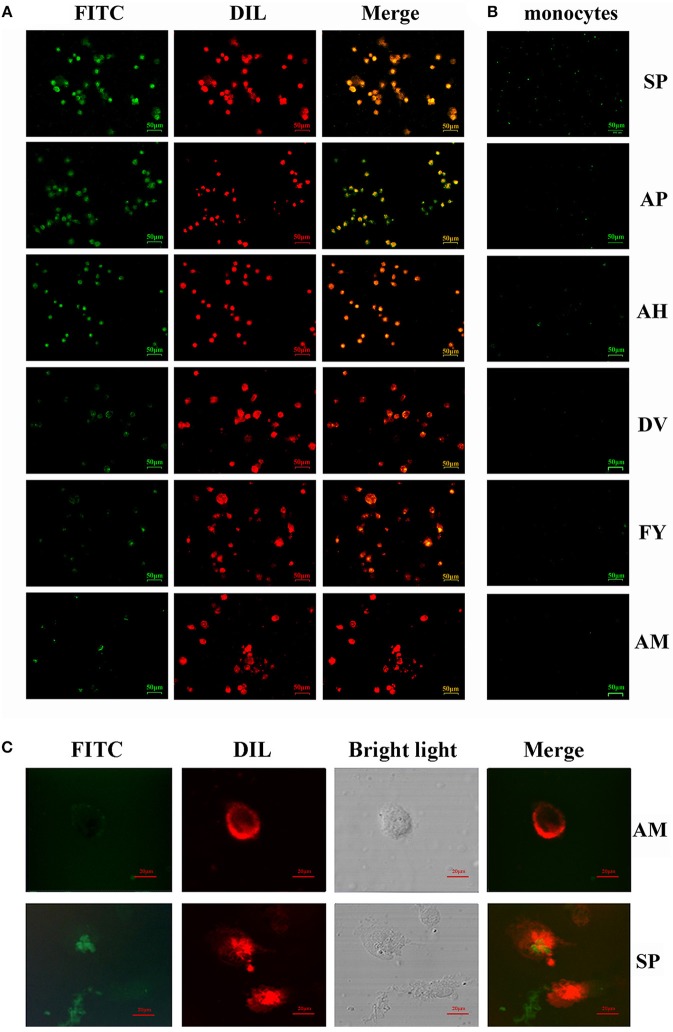
Ability of FITC-labeled peptides to bind to chBM-DCs was analyzed by fluorescence microscope and laser confocal microscopy. Fluorescence analysis of peptides binding to chBM-DCs **(A)** and monocytes **(B)**. The location of FITC-labeled peptides binding to DCs was analyzed by laser confocal microscopy **(C)**. The cell membrane dyed by DIL is shown in red. FITC-labeled peptides are shown in green. Changes to the brightness, contrast or color balance were applied to every pixel in the image by microscopy.

For more accurate quantitative analysis, we used flow cytometry to detect the ability of synthetic FITC- labeled peptides binding to chBM-DCs. The results of flow cytometry (Figure 1B) showed that the counts of FITC-labeled peptides binding to chBM-DCs were close, revealing that the six candidates can attach to DCs under the condition of weak phagocytosis. However, the mean fluorescence intensities (Figure 1C) were strikingly different, which gives prominence to SP. For further verification, we observed the fluorescence of peptides binding to chBM-DCs and monocytes by fluorescence microscopy. Figures 2A,B show that the peptides hardly bound to the monocytes, as expected, and SP, AP, and AH showed better effects. SP can attach to the cell membrane dyed by DIL or be engulfed by chBM-DCs and be shown as yellow when merged (Figure 2C). To summarize, SP had the best binding ability among the six candidates and was finally chosen as the targeting ligand for further steps in our study.

### Fusion of SP to VP2 Alters mRNA Levels of Markers and Cytokines of DC

**Figure 3:**
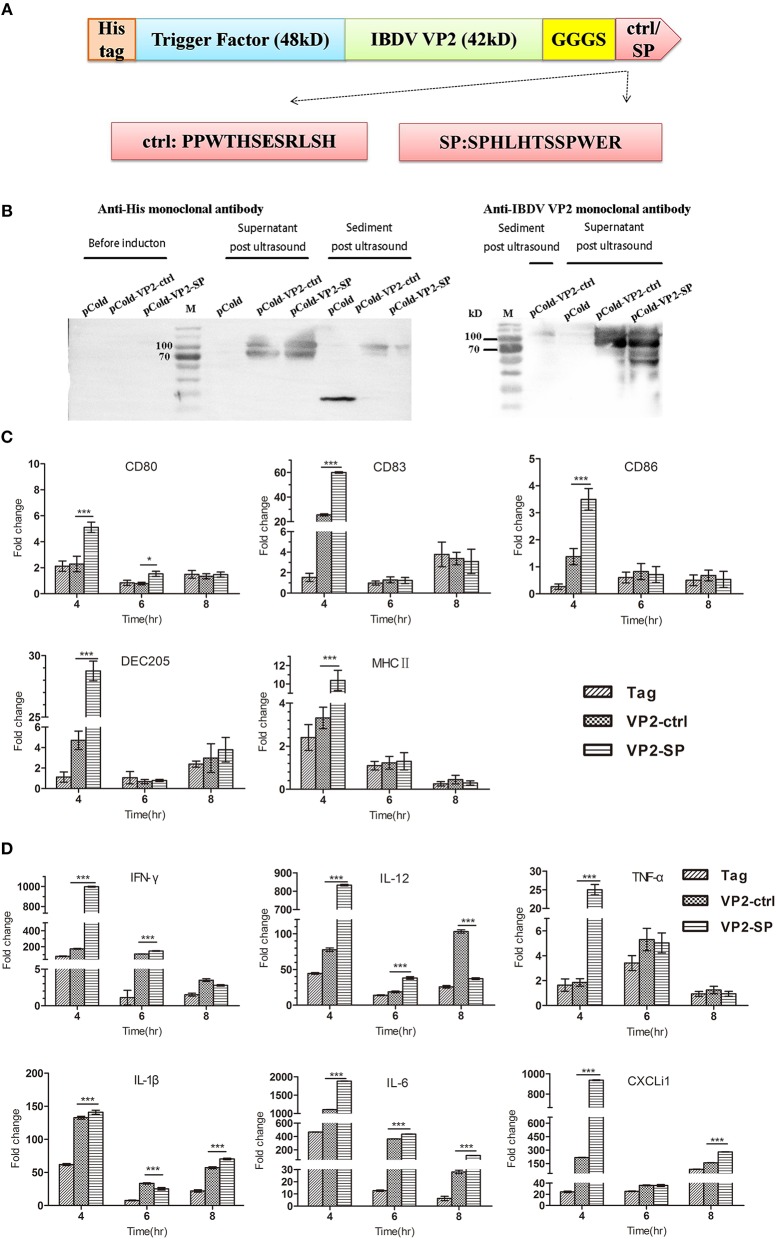
Cytokines expression by chBM-DCs was assessed by relative qRT-PCR. Construction of recombinant vector in *E. coli* **(A)**. Identification of recombinant protein expressed in BL21 by western blot **(B)** (anti-His and IBDV VP2 monoclonal antibody was used as the primary antibody, respectively). See also Supplementary Figure 2. Some chBM-DCs markers expression by chBM-DCs stimulated by VP2-ctrl and VP2-SP was assessed by relative qRT-PCR **(C)**. Cytokines expression by chBM-DCs stimulated by VP2-ctrl and VP2-SP was assessed by relative qRT-PCR **(D)**. *p < 0.05, ***p < 0.001.

We next tested the mRNA of markers expressed by chBM-DCs and cytokines when VP2 was genetically fused to SP or the control peptide. First, we constructed recombinant pCold plasmid in *E coli*. Figures 3A,B show that recombinant protein (about 90 kD) can be detected by western blotting and that it was mainly soluble. Purified VP2-SP and VP2-ctrl were used for stimulating chBM-DCs *in vitro*. Non-stimulated chBM-DCs at the same point were used as calibrator, and β-actin was chosen as the reference gene (data not shown). The up-regulation of chBM-DCs marker genes such as costimulatory molecules and MHCII indicates the maturation of DCs. Figure 3C shows that CD80, CD83, CD86, DEC205, and MHCII expressed by VP2-SP-stimulated chBM-DCs were significantly higher than in the VP2-ctrl group at 4 h (*p* < 0.001). However, with the prolongation of incubation time, at 6 and 8 h, there was almost no difference between the VP2-SP group and VP2-ctrl group (*p* > 0.05), except for CD80 (6 h).

The cytokines released by DCs are involved in immune responses and have a critical effect on differentiation of naïve T cells into Th1 and Th2. Figure 3D shows that the mRNA levels of Th1-associated cytokines (IFN-γ and IL-12), were significantly higher in the VP2-SP groups than in the VP2-ctrl groups at 4 h. IFN-γ has antiviral activities and can up-regulate the expression of MHC antigens. IL-12 plays an important role in the activities of natural killer cells and T lymphocytes. Pro-inflammatory cytokines (TNF-α, IL-1β, and IL-6), and chemokines (CXCLi1) mRNA levels were also higher in the VP2-SP groups than in the VP2-ctrl groups at 4 h. However, at 8 h for IL-12 and at 6 h for IL-1β, the levels of VP2-control groups were higher than those of the VP2-SP groups. Based on these results, we conclude that SP can direct the antigen VP2 to chBM-DCs, at least at the time point of 4 h.

### Expression of the Fused VP2-SP in *L. saerimneri*

**Figure 4:**
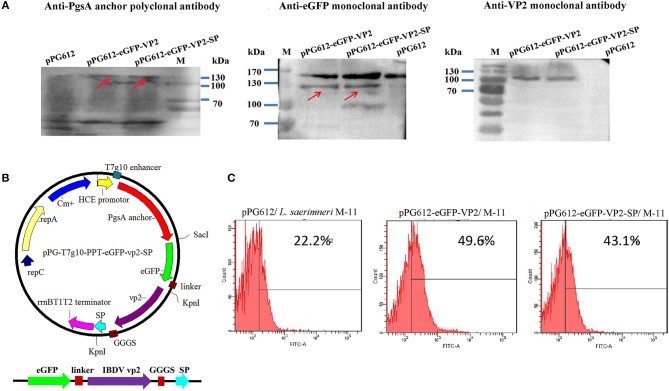
Identification of heterologous protein expressed by *L. sae M-11*. Identification of recombinant VP2 protein expressed in *L. sae* M-11 through western blotting (anchor polyclonal antibody, anti-eGFP monoclonal antibody, and anti-IBDV VP2 monoclonal antibody were used as the primary antibodies) **(A)**. The calculated molecular weight of the fused protein was about 132kD. PgsA anchor, eGFP and VP2 were about 63, 27, and 42 kD, respectively. See also Supplementary Figure 2. Construction of the recombinant plasmid pPG-T7g10-eGFP-PPT-VP2-SP **(B)**. Identification of recombinant protein expression in *L. sae* M-11 through flow cytometry **(C)**.

To evaluate immunogenicity *in vivo*, we established an oral vaccine delivery system. First, the recombinant plasmids (Figure 4B) were transformed into *Lactobacillus*. Then, the expression of the recombinant protein was determined by western blotting. As shown in Figure 4A, the fusion protein was about 130 kDa, consistent with the predicted molecular weight (pgsA anchor, about 63kD, eGFP, about 27 kD, VP2 about 42kD). The negative control, pPG612, did not express a corresponding immunoreactive band.

Flow cytometry was used to determine eGFP expression in recombinant *Lactobacillus* quantitatively. Figure 4C shows that in the early logarithmic phase of the bacteria, green fluorescence could be detected in the pPG612-eGFP-VP2/M-11 and pPG612-eGFP-VP2-SP/M-11 compared with pPG612/M-11, and this proved that eGFP expressed in *L. saerimneri* had bioactivity. The amount of bacteria expressing heterologous protein was approximately 20%. Therefore, the protein of interest has been expressed in *Lactobacillus*, respectively.

### Immune Responses Induced in Chickens by Oral Administration of the Recombinant Strains

**Figure 5:**
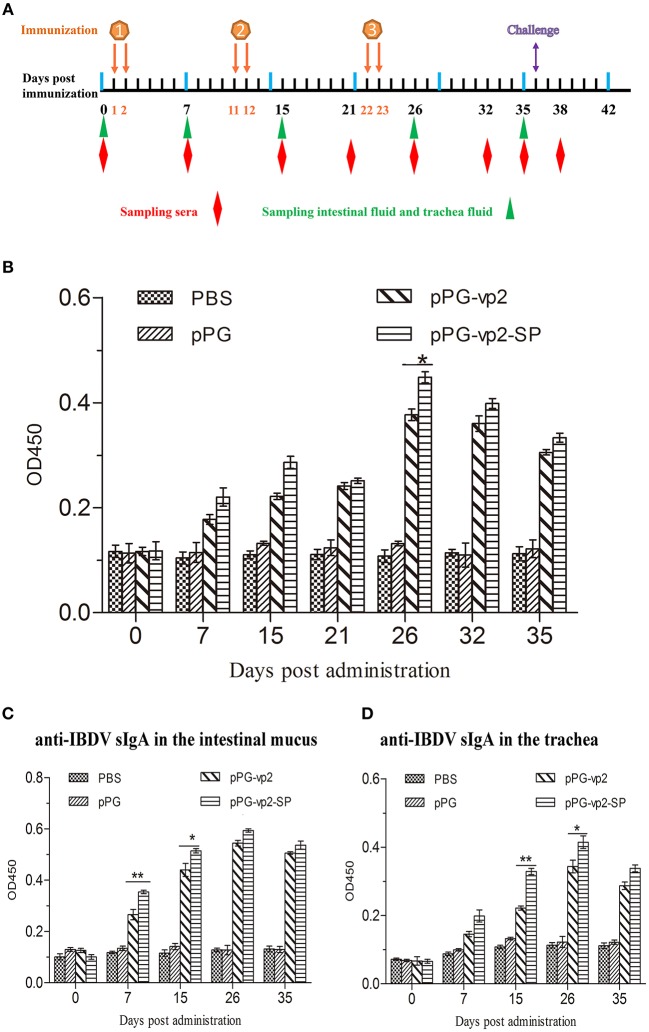
Detection of anti-IBDV-specific IgY and sIgA antibody levels. Scheme of oral immunization and sampling of sera, intestinal mucus, and trachea fluid **(A)**. Primary immunizations were carried out on two consecutive days (days 1 and 2). Booster immunizations were carried out on days 11, 12, 22, and 23. Anti-IBDV-specific IgY antibody levels in the sera **(B)**. Groups (*n* = 4) of SPF chickens were orally immunized with recombinant strains and PBS. Anti-IBDV-specific sIgA levels in the intestinal mucus **(C)** and trachea fluid **(D)** post-immunization with recombinant strains. Bars represent the mean ± standard error value of each group (*p < 0.05, **p < 0.01).

The immunogenicity of the recombinant *Lactobacillus* strains in chickens after oral immunization was evaluated. The systemic and mucosal immune responses were assessed by detecting anti-IBDV IgY and sIgA antibodies by ELISA, respectively. IgY, known as egg yolk immunoglobulin, is one of the predominant class of serum immunoglobulin in birds. SIgA can protect the host by binding to the surface of luminal microbes. Both two antibodies can facilitate specific immune responses. The scheme of oral immunization and specimen collection is shown in Figure 5A. Our results showed significantly higher levels of IBDV-specific systemic IgY antibodies (Figure 5B) on day 26 (*p* < 0.05) and those of IBDV-specific mucosal sIgA antibodies in the intestinal mucus (Figure 5C) on day 7 (*p* < 0.01), and day 15 (*p* < 0.05) post-immunization in chicken orally administered with pPG-vp2-SP, compared with the pPG-vp2 group. The same comparative results were observed using sIgA antibodies in the trachea, day 15 (*p* < 0.01), and day 26 (*p* < 0.05) post-immunization (Figure 5D). These results suggested that SP can accelerate the production of antibodies. However, the difference was not permanent and usually appeared at an early stage after immunization.

The neutralizing activities of antibodies have been widely used in immunotherapeutic applications. The neutralizing activity of sera from chickens orally administered with the recombinant *L. sae* was evaluated by diluting antibodies and mixing with a maintained dose of the virus. The TCID50 of the IBDV cell-adapted strain in the DF-1 cells was measured and calculated by the Reed-Muench method (about 10^−6.1^/ml). The serum-neutralizing antibody titers of the pPG-vp2 group were calculated to be 1:4, 1:6, and 1:4, and titers in the pPG-vp2-SP group were 1:6, 1:8, and 1:8. The titers in the pPG group and PBS group were all <1:2, indicating no neutralizing activity was observed in the sera.

### Induction of Cytokines

**Figure 6:**
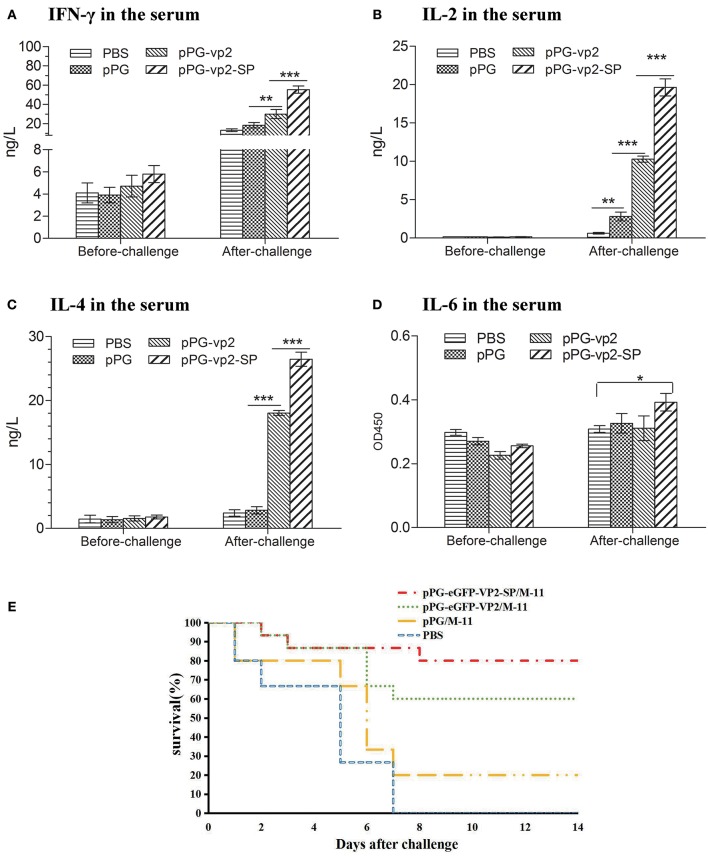
Cytokine levels of sera from SPF chickens **(A–D)** and chicken survival after challenge **(E)**. Cytokines released into the blood of SPF chickens that were bled before and after challenge. Gamma interferon (IFN-γ) **(A)**, interleukin-2 (IL-2) **(B)**, interleukin-4 (IL-4) **(C)**, and interleukin-6 (IL-6) **(D)** were detected. Bars represent the mean ± standard error value of each group (*p < 0.05, **p < 0.01, ***p < 0.001).

**Figure 7:**
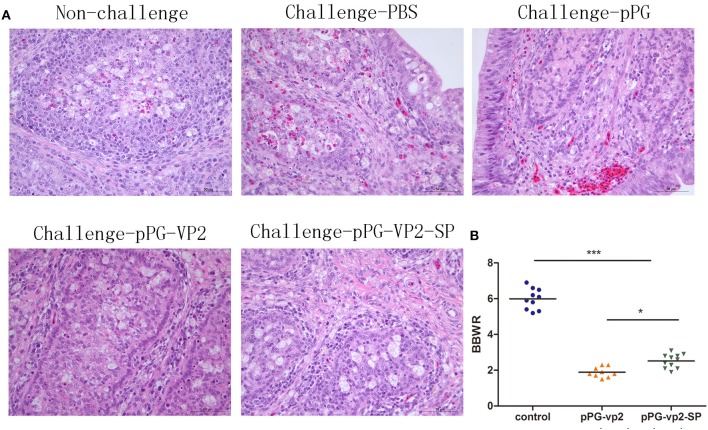
HE staining of SPF chicken Fabricius **(A)** and bursa of Fabricius/body weight ratios **(B)**. Bursa Fabricius to body weight (BBWR) was calculated by (bursal weight/body weight) × 1,000 (*p < 0.05, ***p < 0.001).

Cytokines (IFN-γ, IL-2, IL-4, and IL-6) released into the sera were assayed by ELISA. The results in **Figure 7** show that there was almost no significant difference among four groups before challenge. Figure 6A shows that after challenge, IFN-γ, which has inherent antiviral activity and immunomodulatory effects, was up-regulated significantly compared with that before challenge, and IFN-γ in the vp2-SP group was higher than that in the vp2 group. The same comparative results were observed for IL-4 which is a key regulator in humoral and adaptive immunity (Figure 6C). IL-2 plays crucial roles in regulating both immune activation and homeostasis. In Figure 6B, IL-2 levels were significantly different in each group after challenge with a trend of pPG-vp2-SP > pPG-vp2 > pPG > PBS. The IL-6 level shown in Figure 6D was lower than the detectable threshold of the ELISA kit, but IL-6 in the pPG-vp2-SP group was higher than that in the PBS group.

### Protection Efficiency and Histopathological Results

A challenge experiment was performed using highly virulent IBDV to evaluate the protection efficiency of genetically engineered *Lactobacillus* strains. Loss of appetite and depression were caused in most chickens on day 3 post-challenge. In the next few days, white watery stools occurred, especially in the PBS group. Results in chickens given two booster immunizations showed that both pPG-eGFP-vp2-SP/M-11 and pPG-eGFP-vp2/M-11 had good protective effects with a rate of 80% (12 chickens) and 60% (9 chickens), respectively. By contrast, chickens in the PBS group developed severe clinical signs of infection and died by 1 week post-challenge (Figure 6E). Twenty percent of the chickens in the pPG/M-11 group survived with mental depression and reduction in food consumption.

IBDV replication often leads to extensive lymphoid cell destruction in the bursa of Fabricius. Hematoxylin and eosin staining of the bursa of Fabricius on the 7th day after challenge showed that bleeding points in the vaccine groups were less than those in the pPG/ M-11 group and PBS group (Figure 7A). However, follicular atrophy, massive necrosis of lymphocytes, connective tissue hyperplasia, and macrophage infiltration existed in the vaccine groups, the pPG/ M-11 group, and the PBS group. Chickens which survived the acute phase of the disease recovered from clinical disease. Figure 7B shows the shrinking of the bursa that appeared in all the surviving chickens on the 14th day post-challenge. Interestingly, the shrinking in the pPG-eGFP-vp2-SP/M-11 group was less than that in the pPG-eGFP-vp2/M-11 group (*p* < 0.05).

**Figure 8:**
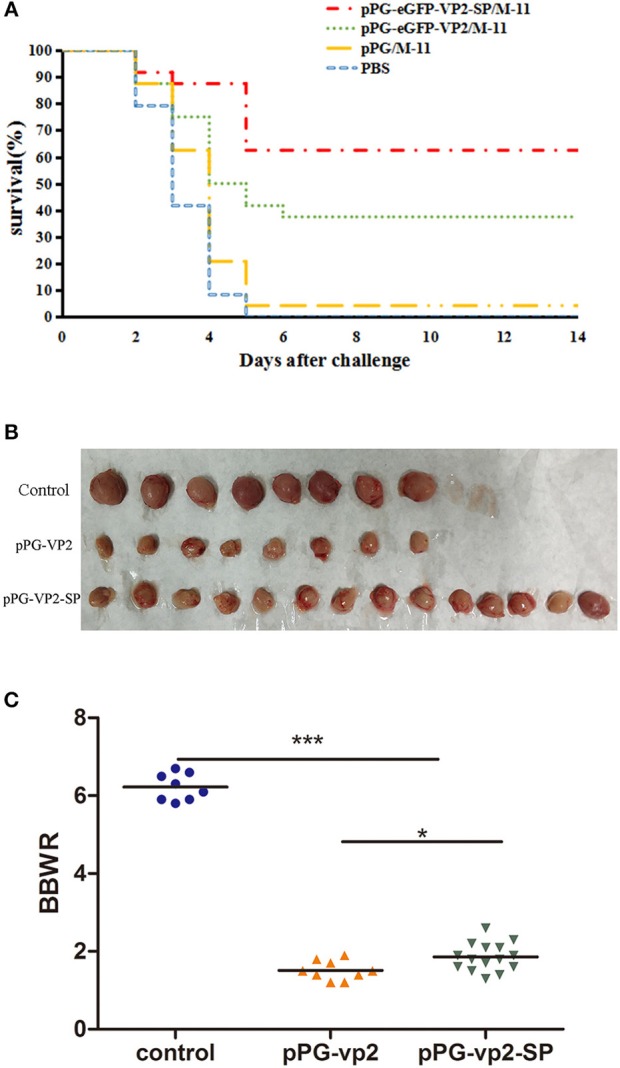
Broiler chicken survival after challenge **(A)**, bursa Fabricius in surviving chickens **(B)**, and bursa Fabricius/body weight ratios (*p < 0.05, ***p < 0.001) **(C)**. Broiler chickens (24 per group) were orally challenged with very virulent IBDV at 22 days post-immunization.

Results in chickens with one booster immunization (Figure 8A) showed that both pPG-eGFP-vp2-SP/M-11 and pPG-eGFP-vp2/M-11 had a good protective effect with rates of 62.5% (15 chickens) and 37.5% (9 chickens), respectively. Figure 8B shows the bursa in each group. Figure 8C shows that the shrinkage in the pPG-eGFP-vp2-SP/M-11 group was less than that in the pPG-eGFP-vp2/M-11 group (*p* < 0.05). From the above results, it can be concluded that, with the participation of SP, the protection efficiency increased.

## Discussion

In recent years, the DC-targeting strategy has become one of the focuses in research as DCs play a significant role in the induction and regulation of immune responses. Much progress has been made in human DC-targeting vaccines. This strategy can increase the number of antigens targeting DCs. As a result, vaccine doses can be decreased appropriately to induce strong and rapid immune responses, simultaneously (28–30). As poultry vaccines should take practicability into consideration, including production cost, and wide-scale administration, DC-targeting vaccines have become a desirable choice (31). Hence, we used *Lactobacillus* to deliver antigens via chBM-DC-targeting peptides into DCs.

Some symbiotic gut microbes are predicted to evolve host-specific adaptations (32–34). Therefore, *L. sae* M-11 with good colonization, especially in chicken cecum, which is close to the cecum tonsil, was applied as the delivery vector. Theoretically, recombinant *L. sae* M-11 can adhere to the intestinal epithelium to compete with pathogenic bacteria for binding epitopes and propagate in the chicken intestine. In fact, only a small quantity of immunogen of interest can penetrate the gut wall to be taken up by APCs (35). To increase the bioavailability of the immunogen in chickens, the DC-targeting peptide was screened in this study. Binding peptides were obtained through the phage display library, which has been used in screening the binding peptides of proteins, cells, or organs *in vivo* (36–38). However, there was no obvious enrichment after four rounds of screening because of the complex components of the cell surface. Although peptides are displayed randomly in the original phage library, the frequency of each amino acid is different. For these reasons, we used various experiments to verify that the peptide named SP has more potential for targeting chBM-DCs. Of these, antigen fused with SP can regulate markers of chBM-DCs and some cytokines at early times *in vitro*, and this implied that SP can direct VP2 to DCs. Then, a live recombinant *Lactobacillus* vaccine with SP was constructed to detect whether the targeting peptide can work in the chicken intestinal tract.

Ideally, DCpep helps antigens to be caught by DCs, which are located in or beneath the epithelium. Antigens can be processed and presented by DCs to lymphocytes directly or migrate into other lymphoid tissue for antigen presentation. B cells are activated to secrete sIgA, T cells differentiate, and then protective immunity is induced (39). T and B cells activated by DCs then move to the periphery to trigger a specific immune response against the pathogen challenge (40). This study indicated that compared with that induced by the pPG-VP2 group, recombinant *Lactobacillus* with chBM-DC-targeting peptide can promote more rapid immunity at both mucosal and systemic levels. With the challenge of very *virulent IBDV*, higher Th1/Th2-associated IL-2, Th2-associated IL-4, and Th1-associated IFN-γ in vaccine groups than those in the control group revealed that cellular immunity was initiated. The pPG-VP2-SP group showed a significant protective effect according to the challenge test and gross lesions in the bursa. These results indicate that this approach may be useful for avian preventive vaccinations.

The avian mucosal immune system can supervise pathogens properly and be tolerant to commensal microbes together with dietary antigens (41, 42). Correspondingly, mucosal antigens are usually less immunogenic compared with those delivered by other routes (43, 44). Potential adjuvant and delivery systems are needed for effective mucosal vaccination (45, 46). However, inadequate research on avian immunity has hampered the development of avian mucosal vaccines. Our study showed that the peptide named SP derived from a phage display peptide library can bind to chBM-DCs *in vitro* and assist antigens with promoting systemic and mucosal immune responses via fused expression in a *Lactobacillus* system. Further research should identify which receptor on the DCs was targeted and relevant mechanisms of initiating desired immune responses. In addition, to deliver the antigen to chicken APCs efficiently, exploration of various antigen-targeting systems including ligands, antibodies, or nanoparticles is still urgently required.

In this study, the chBM-DCs binding peptide SP was obtained through the Ph.D.-12 phage display library method and identified. *L. sae* M11 was used as an antigen vector to deliver IBDV VP2 fused with SP as an oral vaccine. The results indicated that anti-IBDV mucosal and humoral immune responses were induced efficiently via oral administration, resulting in a higher protective efficacy in the VP2-SP group compared with the VP2 group. Therefore, chicken DCs targeting the IBDV protective antigen VP2 delivered by *L. sae* provide effective immune protection in chickens, prompting it as a potential strategy for the development of a vaccine against IBDV infection.

## Data Availability

All datasets generated for this study are included in the manuscript and/or the Supplementary Files.

## Ethics Statement

Animal experiments were carried out in accordance with the recommendations in the institutional and national guidelines for animal care and use. The protocol was approved by the Committee on the Ethics of Animal Experiments of Northeast Agricultural University, Harbin, China (2016NEFU-315, 13 April 2017).

## Author Contributions

YX and SM designed and conducted research experiments. XH contributed to isolation and validation of chBM-DCs. XQ, XW, and LW performed screening of the targeting peptide by phage display, gene cloning, electrotransformation, and expression characterization (western blot and FACS). HZ, WC, and YJ presided over laboratory animals, performed all immunizations, collected samples, and performed ELISA. YX, LT, YL, and SM analyzed the data and wrote the paper. All authors read and approved the final manuscript.

### Conflict of Interest Statement

The authors declare that the research was conducted in the absence of any commercial or financial relationships that could be construed as a potential conflict of interest.
