# Supplementary material for: Screening and Identification of a Chicken Dendritic Cell Binding Peptide by Using a Phage Display Library
Source: Front Immunol. 2019 Aug 6;10:1853. doi: 10.3389/fimmu.2019.01853 (PMC6691127; doi:10.3389/fimmu.2019.01853)
Supplement: Supplementary file 1 [file Data_Sheet_1.PDF]

Supplementary Material

Table S1. Primers used in real-time PCR.

Primer	Sequence (5′–3′)	GenBank ID
	F: TGCTGTGTTCCCATCTATCG	
β-actin	R: TTGGTGACAATACCGTGTTCA	NM_205518.1
	F:AGCCCAAGGTAAGGGTCTCG	
MHC Ⅱ	R: CGCCCGCTCAGGAACCACT	NM001001762.1
	F: AACAGAAATGGTGGTAAGAT	
CD80	R: TAGAAGCAAACTGGTGGA	NM_001079739.1
	F: TGTGAAGACTGGGTAATAGG	
CD83	R: AGGACAACAGCAGGAAGC	XM_418929
	F: TTGCTACTTCCCAAACTC	
CD86	R: TCATCCACAATCCCTACA	EF554724.1
	F: GCCCTCCTCCAGCCAGAAAG	
IL-1β	R:TAGCCCTTGATGCCCAGTGC	NM_204524.1
	F: TTCACCATCAGGCACGAC	
DEC205	R: AAGGCGACTCTGGGACAC	NM_001037836
	F: GCAGGACGAGATGTGCAA	
IL-6	R: CCAGGTAGGTCTGAAAGGC	AJ309540
	F: R ACCTCTTCAAGGGTGCACTCA	
IL-12	R: ACCTCTTCAAGGGTGCACTCA	AY262751
	F: ATCATACTGAGCCAGATTGTTTCG	
IFN-γ	R: TCTTTCACCTTCTTCACGCCAT	Y07922
	F: GAAGCAGCGTTTGGGAGT	
TNF-α	R: GTTGTGGGACAGGGTAGG	NM_204267.1
	F: TGGCTCTTCTCCTGATCTCAATG	
CXCLi 1	R: GCACTGGCATCGGAGTTCA	Y14971

Table S2. Sequences and frequency of peptides

Name	Sequence	Frequency
SP	SPHLHTSSPWER	16/114 (14.0%)
GD	GDWPSLMHPRLE	5/11 (4.4%)
AP	APSRTQINADYR	5/114 (4.4%)
TP	TPPIRLTDSSAS	4/114 (3.5%)

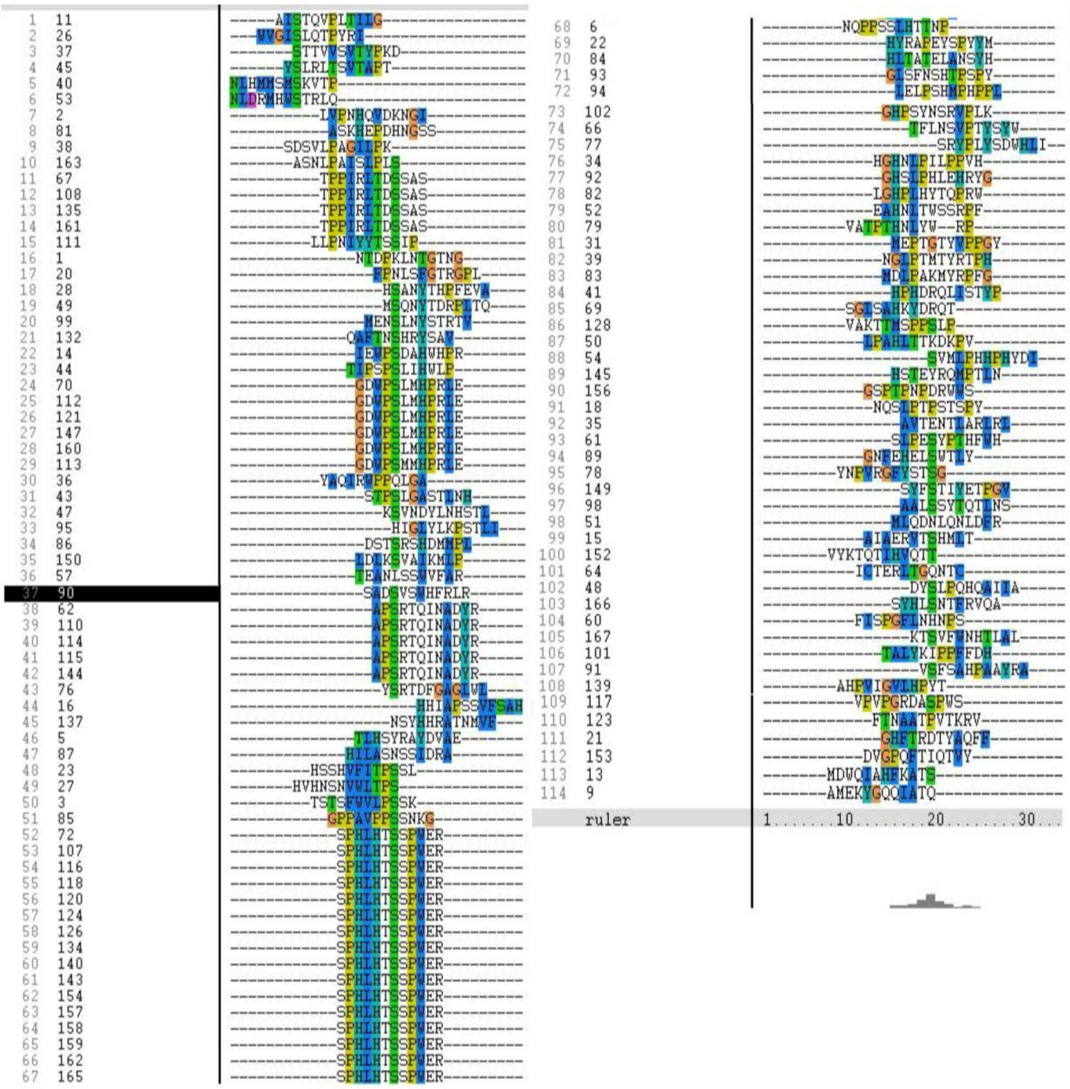

Supplementary Figure 1. Sequences of 12-mer peptides. After the fourth round of biopanning, individual phage-plaques were randomly selected and amplified separately. The nucleotide sequence of each phage was translated to its corresponding peptide sequence.

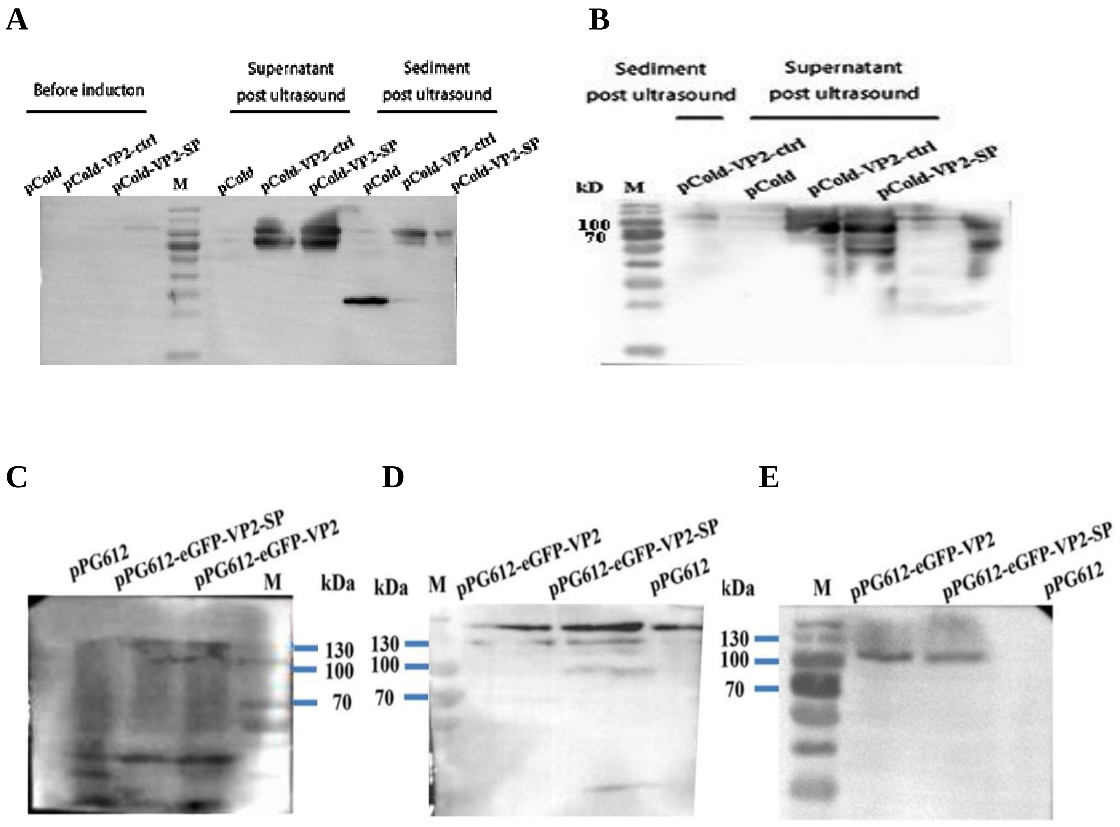

Supplementary Figure 2. Western blot. Identification of recombinant VP2 protein expressed in BL21 by western blot (A and B). Anti-His monoclonal antibody was used as the primary antibody (A). IBDV VP2 monoclonal antibody was used as the primary antibody (B). Identification of recombinant VP2 protein expressed in *L. sae* M-11 through western blotting (C, D and E). Anchor polyclonal antibody was used as the primary antibody (C). Anti-eGFP monoclonal antibody was used as the primary antibody (D). Anti-IBDV VP2 monoclonal antibody was used as the primary antibody (E).
